# Attenuation of Chondrogenic Transformation in Vascular Smooth Muscle by Dietary Quercetin in the MGP-Deficient Mouse Model

**DOI:** 10.1371/journal.pone.0076210

**Published:** 2013-09-30

**Authors:** Kelly E. Beazley, Florence Lima, Teresa Borras, Maria Nurminskaya

**Affiliations:** 1 Department of Biochemistry and Molecular Biology, School of Medicine, University of Maryland, Baltimore, Maryland, United States of America; 2 Department of Ophthalmology, School of Medicine, University of North Carolina, Chapel Hill, North Carolina, United States of America; Center for Cancer Research, National Cancer Institute, United States of America

## Abstract

**Rationale:**

Cartilaginous metaplasia of vascular smooth muscle (VSM) is characteristic for arterial calcification in diabetes and uremia and in the background of genetic alterations in matrix Gla protein (MGP). A better understanding of the molecular details of this process is critical for the development of novel therapeutic approaches to VSM transformation and arterial calcification.

**Objective:**

This study aimed to identify the effects of bioflavonoid quercetin on chondrogenic transformation and calcification of VSM in the MGP-null mouse model and upon TGF-β3 stimulation in vitro, and to characterize the associated alterations in cell signaling.

**Methods and Results:**

Molecular analysis revealed activation of β-catenin signaling in cartilaginous metaplasia in *Mgp-/*- aortae in vivo and during chondrogenic transformation of VSMCs in vitro. Quercetin intercepted chondrogenic transformation of VSM and blocked activation of β-catenin both in vivo and in vitro. Although dietary quercetin drastically attenuated calcifying cartilaginous metaplasia in *Mgp-/*- animals, approximately one-half of total vascular calcium mineral remained as depositions along elastic lamellae.

**Conclusion:**

Quercetin is potent in preventing VSM chondrogenic transformation caused by diverse stimuli. Combined with the demonstrated efficiency of dietary quercetin in preventing ectopic chondrogenesis in the MGP-null vasculature, these findings indicate a potentially broad therapeutic applicability of this safe for human consumption bioflavonoid in the therapy of cardiovascular conditions linked to cartilaginous metaplasia of VSM. Elastocalcinosis is a major component of MGP-null vascular disease and is controlled by a mechanism different from chondrogenic transformation of VSM and not sensitive to quercetin.

## Introduction

In the normal vasculature, the principal function of smooth muscle cells is contraction to regulate blood pressure and blood flow throughout the body [1]. In contrast to the other terminally-differentiated muscle cells, cardiac and skeletal, vascular smooth muscle cells (VSMCs) undergo a unique phenotypic modulation during normal development, and in the physiologic response to blood vessel remodeling or injury. This is associated with decreased expression of VSMC-specific contractile genes and increased cell proliferation and migration. However, there is growing evidence that this phenotypic plasticity of VSMCs contributes to vascular disease by allowing for differentiation to inappropriate lineages [1–3]. For example, in diabetic arteriosclerosis VSMCs undergo osteogenic transformation, losing contractile gene expression and depositing a calcified bone-like matrix. Together this results in increased vessel stiffness and decreased compliance, a strong predictor of cardiovascular risk [4].

Recent mechanistic studies reveal the complexity of phenotypic instability in VSMCs. The literature in this field is extensive and identifies diverse regulators including, but not limited to, growth factors, inflammatory cytokines, calcium-phosphate homeostasis, oxidized phospholipids, retinoic acid and mechanical stress, and involves multiple signaling pathways including MAPK kinases, Rho, Notch, BMP and β-catenin signaling (reviewed in [5–7]).

Lineage tracing studies have demonstrated that VSMC-derived hyperproliferative cells play a role in neointimal formation induced by either injury [8] or atherosclerosis [9], and that osteochondrogenic transformation of VSMCs associates with calcification of the arterial media [10–13] as well as atherosclerotic calcification [14]. Vascular calcification (or pathologic calcium phosphate deposition in the blood vessels) is a common complication associated with diabetes, hypercholesterolemia, chronic renal insufficiency and osteoporosis, and is highly correlated with cardiovascular disease mortality. Its clinical consequences include stroke, amputation, ischemic heart disease, and complications during artery stenting. In addition, vascular calcification is now recognized as a marker of atherosclerotic plaque burden [15,16]. The incidence of vascular calcification also correlates with age, putting a growing percentage of our population at risk as life expectancy increases.

A decrease in circulating and local inhibitors of mineralization, especially in dialysis patients with chronic kidney disease, appears to significantly contribute to calcification. One of the major anti-calcific factors is matrix Gla protein (MGP), a 10 kDa polypeptide containing calcium-binding γ-carboxyglutamic acid (Gla) residues and phosphoserine residues. In the vasculature, MGP is expressed in vascular smooth muscle (VSM) and endothelium [17,18]. MGP polymorphisms have been linked to coronary artery calcification in patients [19,20], while in mice genetic loss of MGP leads to cartilaginous metaplasia and extensive calcification of the tunica media [21]. MGP-null arterial disease has been described primarily as a product of chondrogenic transformation of *Mgp-/-* VSM resulting in the ectopic formation of cartilage in the vessel wall, extensive calcification, and ultimately rupture of the blood vessels soon after birth [21]. While mechanisms underlying the osteogenic transformation of VSM induced by warfarin [10–13] and by hyperphosphatemia [22,23] are emerging [7,24,25], the chondrogenic transformation of vascular cells that is prominent in uremia- and diabetes-associated medial calcification [26,27] is not well understood. The *Mgp-/-* vasculature is an excellent model for mechanistic analysis of chondrogenic transformation in VSM because 97% of the chondrocyte-like cells in arterial cartilaginous metaplasia originate from VSM [13] suggesting a minimal (if any) contribution by circulating or resident multipotent mesenchymal progenitors.

With a growing mechanistic understanding, treatment options for vascular diseases are rapidly developing. In particular, emerging interest has been focused on the protective effects of flavonoid-rich diets in cardiovascular disease. The reported beneficial effects of the major bioflavonoid, dietary quercetin, in humans include lower blood pressure and LDL levels and overall reduced cardiovascular disease-related mortality [26–29]. In animal models, quercetin effectively alleviated atherosclerosis [30,31] and attenuated warfarin-induced hypertension and elastocalcinosis [12]. Quercetin has anti-inflammatory, anti-oxidative and anti-proliferation effects. In addition, we have found that in VSMCs quercetin inhibits β-catenin signaling, central for osteogenic transformation of these cells, and vascular calcification induced by warfarin [12,25]. β-catenin is a multi-tasking molecule regulating developmental and homeostatic processes. It is an integral part of adherent junctions and the key mediator of the canonical Wnt/β-catenin signaling cascade, and has been shown to cross-talk directly with non-Wnt pathways [32]. During development Wnt/β-catenin signaling plays a manifold role and promotes physiological chondrocyte maturation [33] as well as vascular remodeling and differentiation [34]. In adult vessels, the β-catenin pathway is usually dormant but activates in disease [35]. In particular, a critical role for β-catenin signaling has been shown in warfarin-induced calcification [36,37]. Further, Wnt/β-catenin signaling has also been implicated in BMP2-induced aortic mineralization in the diabetic *LDLR-/-* mice [38] and in calcification of heart valves [39]. Here, we investigated β-catenin signaling associated with chondrogenic transformation of *Mgp-/-* VSM, and examined the efficiency of quercetin in alleviating the MGP-null vascular disease.

## Materials and Methods

### Animals

4.5 to 5 week-old C57BL/6J wild-type mice (*Mgp+/+*) and mice deficient in MGP (*Mgp-/-*

) were used in this study. MGP-deficient mice were a kind gift from Dr. Karsenty, Columbia University, New York. Mice were genotyped using standard PCR for *Mgp*. All procedures were approved by the institutional animal care and use committees at the University of Maryland Medical School (protocol #: 0912010) and the University of North Carolina School of Medicine, and were conducted in compliance with National Institutes of Health guidelines for the care and use of laboratory animals.

### Quercetin treatment

Quercetin (Quercegen, Newton, MA) was administered as a dietary supplement (0.02% w/w in drinking water) to weaned pups for 2 weeks. Alternatively, quercetin was given to lactating Mgp+/- females immediately after they gave birth to the progeny and continued throughout the 3-week breastfeeding period. Weaned Mgp-/- pups continued to receive quercetin in drinking water for an additional 2 weeks.

### Immunohistochemistry and Histological Staining

Frozen 10 µm sections of freshly-dissected aortas fixed in 4% paraformaldehyde were stained for proteoglycan deposition and calcified matrix using the alcian blue and von Kossa silver nitrate methods, respectively, according to standard protocols [40].

Immunofluorescence was performed using the standard protocol. Briefly, tissue was blocked with 10% goat serum for 30 minutes at room temperature. Tissue sections were incubated with primary antibodies diluted in 1% goat serum overnight at 4°C and secondary antibodies diluted in PBS for 1.5 hours at room temperature. Nuclei were counterstained with 4',6-diamidino-2-phenylindole (DAPI). Primary antibodies used were rabbit anti-β-catenin (1:80; Santa Cruz Biotechnology) and rabbit anti-Ki67 (1:100; Abcam), detected with a secondary Dylight 549-conjugated goat anti-rabbit antibody (1:400; Jackson ImmunoResearch); mouse anti-type II collagen antibody (a kind gift of Dr. Thomas Linsenmayer), detected with a secondary Alexa 488-conjugated goat anti-mouse antibody; and mouse anti-smooth muscle actin antibody directly conjugated to fluorescein isothiocyanate (FITC). Images were collected using a Leica DMIL inverted microscope equipped with a SPOT RT3 real-time CCD camera (Diagnostic Instruments).

### Morphologic Analysis

For morphologic analyses, serial sections spaced 100 µm apart along a 1 mm length of descending aorta from heterozygous (*Mgp+/-*

), *Mgp-/-*, and quercetin-treated *Mgp-/-* (*Mgp-/-* +Querc) animals were analyzed for chondrogenic lesions and average thickness of tunica media. For each animal, the average value of 4-5 serial sections was used. Mean and standard error values used for graphical display were calculated from the average values of each animal. All values were normalized to wild-type animals (set at 100%).

### Cell culture and Luciferase Analysis

Murine aortic smooth muscle cells were obtained by a modification of the explant method described by Ross [41]. Briefly, medial tissue was isolated from segments of thoracic aorta from wild-type C57BL/6J (*Mgp+/+*) mice. Small pieces of tissue (1 to 2 mm) were loosened by a 1-hour incubation at 37°C in medium supplemented with 165 U/mL collagenase type I. Partially digested tissues were placed in 6-well plates and cultured for 10 days in DMEM supplemented with 20% FBS at 37°C in a humidified atmosphere containing 5% CO_2_. Cells that migrated from the explants were collected and maintained in growth medium (DMEM containing 10% FBS and 100 U/mL of penicillin and 100 µg/mL of streptomycin). To confirm that the cells isolated were VSMCs, α-smooth muscle actin expression was confirmed by immunofluorescence (data not shown). For experiments, cells were used at passages 2 and 3.

The A10 clonal embryonic rat aortic smooth muscle cell line (ATCC, Manassas VA) was also maintained in growth medium. A stable β-catenin-dependent luciferase reporter cell line was established by transducing A10 VSMCs with the Cignal Lentiviral TCF/LEF luciferase reporter (SA Biosciences), according to manufacturer’s protocol, followed by a 2-week selection with puromycin (10 µg/mL). Luciferase activity in whole cell lysates was measured in a 96-well plate luminometer (Harta Instruments, Bethesda MD) using the Promega Luciferase Assay Kit and was normalized to the total lactate dehydrogenase (LDH) present in whole cell lysates, measured using a commercial LDH activity kit (BioVision, San Francisco CA). LDH activity in the culture medium was also measured to determine cell viability.

To induce chondrogenic transformation, VSMCs were seeded at high density (2.5x10^5^ cells in 10 µL volume) and cultured in chondrogenic medium (ChoM) [DMEM–high glucose supplemented with 10^−7^ mmol/L dexamethasone, 0.1 mmol/L ascorbic acid (Wako Chemicals, VA), 1% ITS premix (BD Biosciences, NJ), 1 mmol/L sodium pyruvate, 0.35 mmol/L L-proline, 4 mmol/L L-Glutamine (Invitrogen), 1% Pencillin–Streptomycin (Invitrogen), and 10 ng/mL TGF-β3 (ProSpec, NJ)] in the presence or absence of quercetin (50 µmol/L) or recombinant Dkk1 (0.5 µg/mL; R&D Systems) at 37°C, 5% CO2. Medium was changed twice a week for up to 14 days. Sulfated glycosaminoglycan (GAG) synthesized by cultured VSMCs was detected by fixing them in 4% PFA and staining with 1% Alcian blue (8GX) dissolved in 0.1 N HCl. For quantitative analysis, Alcian blue was extracted with 4 M guanidine hydrochloride and absorbance at 590 nm was measured using an Optima spectrophotometer (PolarStar). Cell density was estimated using WST substrate (Dojindo).

### qRT-PCR

mRNA was isolated using the RNeasy micro kit (Qiagen) and reverse transcription was performed with MaximaRT kit (Thermo, Fisher). Quantitative real-time PCR was performed according to the standard protocol with EVA green chemistry in a CFX96 thermocycler (Bio-Rad) using the primers in Table 1. Relative change in gene expression was calculated by the ΔΔCt method using Microsoft Excel.

**Table 1:** Primer sequences for mouse genes analyzed by real-time PCR.

**Gene**		**Accession #**	**Forward primer**	**Reverse primer**
***β-catenin targets***				
Lrp6		NM_008514	ggtgtcaaagaagcctctgc	gctcgaggactgtcaaggtc
Tcf4		NM_013685	cgaaaagttcctccgggtttg	cgtagccgggctgattcat
Numb		NM_010949	cttcccagttaagtacctcggc	cccgtttttccaaagaagcct
CyclinD1 (cycD1)		NM_007631	gcgtaccctgacaccaatctc	ctcctcttcgcacttctgctc
***Chondrogenic markers***				
Sox9	NM_011448		gttgtaacaccagcagcgtcaag	tgacatactccactttggccacct
Type II collagen (Col II)	NM_031163		caccgaaagtttaagcacaccca	aaataaccctgcccacactcttg
Aggrecan (Agg)	NM_007424		atgccacaagtcacagaaaccacg	aaggcagtcacagcattgttgagc
Indian hedgehog (Ihh)	NM_010544		cgtgaccgaaataagtatggact	gctgctggttctgtatgattg
MMP13	NM_008607		tggagtgcctgatgtgggtgaata	tggtgtcacatcagaccagacctt
TG2	NM_009373		aggtgtccctgaagaacccacttt	ttccacagacttctgctccttggt
***Housekeeping genes***				
Ribosomal protein L19 (Rpl19)	NM_009078		aagaggaagggtactgccaatgct	tgaccttcaggtacaggctgtgat
beta-actin	NM_007393		taatttctgaatggcccaggtct	ctggctgcctcaacacctcaa

### Calcium Determination

Aortas were dried at 55°C overnight and then dissolved in 0.1N hydrochloric acid (HCl) overnight. Calcium content was determined biochemically using the Calcium (CPC) LiquiColor kit as measured with o-Cresolphthalein (Stanbio). Calcium content of the aorta was normalized to dry weight of the tissue prior to HCl extraction.

### Tissue Preparation for Gene Expression Analysis using RT^2^ Profiler PCR Arrays

Aortic tissue from 5 week old mice was disrupted by rapid agitation in the presence of a stainless steel bead (5 mm mean diameter) and lysis buffer using a TissueLyser (2 min at 20Hz, twice; Qiagen). Total RNA was isolated following the RNeasy Fibrous Tissue micro kit protocol (Qiagen) according to the to the manufacturer’s instructions and quantified with NanoDrop spectrophotometer (Thermo Scientific) prior to storage at −80°C. Reverse transcription was performed using 1 µg of RNA according to SA Biosciences’ recommendations using the RT2 First Strand kit. Gene expression analysis was done using the WNT Signaling Pathway PCR Array (PAMM-043), WNT Signaling Targets PCR Array (PAMM-243) and Osteogenesis PCR Array (PAMM-026). ΔΔCt based fold-change calculations were performed using the RT² Profiler PCR Array Data Analysis Template v3.2. In this software standard deviation is calculated for Ct values and not for final fold-changes in expression.

### Statistical Analysis

The data are presented as mean ± standard error. For experiments containing only 2 groups, significance was determined by comparison using Wilcoxon-Mann-Whitney test. For experiments containing more than 2 groups, Levene’s test was used to determine equality of variance (homoscedasticity) followed by 1-way analysis of variance (ANOVA) and Tukey-Kramer post-hoc analysis for comparison between groups. A p-value of < 0.05 was considered to be statistically significant. *, p <0.05; **, p < 0.01; ***, p<0.001.

## Results

### Analysis of proliferation in chondroplastic areas of Mgp-/- aortae

**Figure 1:**
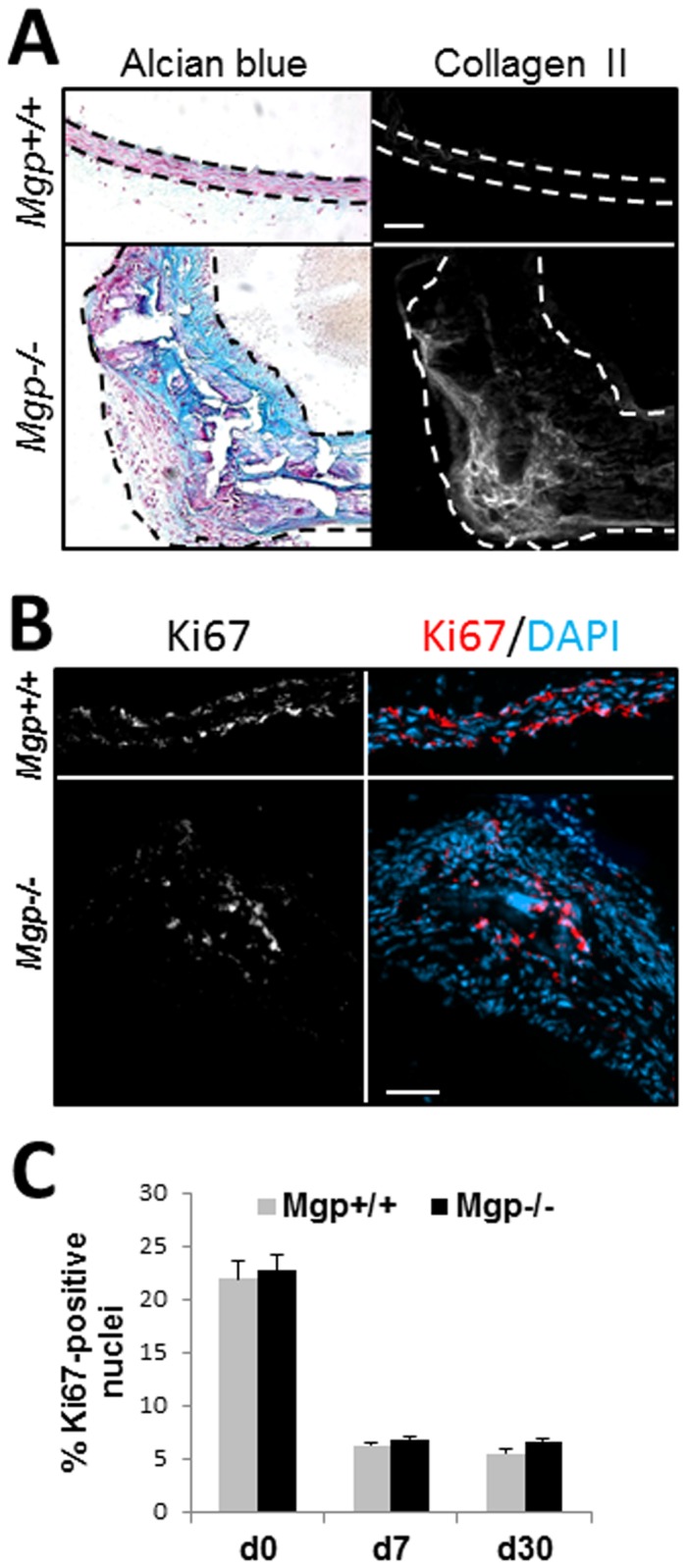
Cartilaginous metaplasia and cell proliferation in the *Mgp-/-* aorta. **A**, Detection of cartilage matrix using Alcian blue staining for GAG deposition and immunostaining for Collagen type II (Collagen II) protein on adjacent sections of aorta from 4.5 week old wild-type (*Mgp+/+*) and *Mgp-/*- animals. Scale = 50 µm. Dashed lines denote internal and external elastic lamina. **B**, Representative immunostaining for cell proliferation marker, Ki67 (white, red) [nuclei counterstained with DAPI (blue)] in 30 day old *Mgp+/+* and *Mgp-/*- aortae. Scale = 50 µm. **C**, Quantitation of the percentage of Ki67-positive nuclei compared to total nuclei in *Mgp+/+* and *Mgp-/*- aortic tissue from animals at birth (d0, N=3), at 7 days old (d7, N=4), and at 30 days old (d30, N=4). Four sections per animal were analyzed.

*Mgp-/-* mice are characterized by the presence of ectopic cartilage in the tunica media [21]. To identify potential mechanisms of the formation of cartilaginous lesions, we first analyzed the possible contribution of increased cell proliferation. Foci of chondrogenic metaplasia were readily detected as areas with the characteristic appearance of rounded chondrocyte-like cells embedded in glycoprotein-rich matrix positive for Alcian Blue staining and cartilaginous collagen type II (Figure 1A), in agreement with previous studies [13,21,42]. We then performed immunohistochemistry for Ki67, a marker of cell proliferation, (Figure 1B) and determined the proportion of the proliferating cells positive for Ki67, compared to total nuclei visible by DAPI nuclear counterstain, in wild-type and *Mgp-/-* aortae. Aortic tissue isolated from newborn, 7- and 30-day old animals was examined. 3 random cross-sections of aortae from each animal were analyzed. The percentage of Ki67-positive proliferating cells was similar between wild-type and *Mgp-/-* aortae at all ages examined (Figure 1C), suggesting that the formation of cartilaginous metaplasia is driven by phenotypic transformation, rather than proliferation, of VSMCs.

### Activation of β-catenin signaling in Mgp-/- aortae

Canonical β-catenin signaling regulates normal physiologic chondrogenesis [33]. Here, we investigated the association between activation of this signaling cascade and chondrogenic transformation in *Mgp-/-* aortae using a qRT-PCR-based microarray of 84 components and regulators of Wnt/β-catenin signaling (4.5 week old Mgp-/- and wild-type mice, N=6). A number of activating Wnt ligands and transcriptional co-activators of β-catenin signaling such as Ccnd3, Pitx2 and Lef1 are induced in the *Mgp-/-* aortae (Table 2), although arterial expression levels of the canonical Wnts detected by the qRT-PCR were very low in both wild-type and *Mgp-/-* tissue (with only Wnt3a threshold cycle number being less than 35). In addition, the drastic down-regulation of sclerostin, an inhibitor of Wnt signaling, further indicates activation of this pathway in *Mgp-/-* arteries (Table 2).

**Table 2:** Changes in expression of genes in the Wnt signaling pathway in *Mgp-/-* compared to wild-type aortae.

**Gene**	**Gene product**	**Fold change**	**p-value**	**Role in Wnt/**β-**catenin signaling**
Ccnd3	Cyclin D3	2.32*	0.021	Co-activator of nuclear β-catenin [56]
Lef1	Lymphoid enhancer binding factor 1	3.63*	0.036	Co-activator of nuclear β-catenin [57]
Pitx2	Paired-like homeodomain transcription factor 2	2.72**	0.009	Co-activator of nuclear β-catenin [58]
Tle2	Transducin-like enhancer of split 2, homolog of Drosophila E(spl)	2.62*	0.014	possible link between Wnt and Notch signaling [59]
Wnt3a	Wingless-related MMTV integration site 3A	2.70*	0.020	
Wnt4	Wingless-related MMTV integration site 4	3.23*	0.028	
***Up-regulated, low abundance (Ct > 35)***				
Fshb	Follicle stimulating hormone beta	2.73**	0.005	transcriptional co-activator for aromatase [60]
Wnt1	Wingless-related MMTV integration site 1	2.35**	0.005	
Wnt10a	Wingless related MMTV integration site 10a	2.35**	0.005	
Wnt3	Wingless-related MMTV integration site 3	2.41*	0.031	
Wnt7a	Wingless-related MMTV integration site 7A	2.35**	0.005	
Wnt7b	Wingless-related MMTV integration site 7B	2.73**	0.005	
Wnt8a	Wingless-related MMTV integration site 8A	3.08*	0.031	
***Down-regulated genes***				
Fzd2	Frizzled homolog 2 (Drosophila)	-2.85*	0.011	
Sost	Sclerostin	-165.46***	0.006	Negative regulator of Wnt signaling [61]
Wif1	Wnt inhibitory factor 1	-8.56*	0.013	
Wnt16	Wingless-related MMTV integration site 16	-17.98***	0.001	non-canonical Wnt signaling, possible link to Notch signaling [62]

Aortic tissue from 4.5 week old animals was analyzed using a qRT-PCR-based microarray N=6. * p<0.05; ** p<0.01; *** p<0.001.

To address this possibility, we used the qRT-PCR-based array approach to study expression of 84 β-catenin target genes in *Mgp-/-* and wild-type aortae (N=6). This analysis revealed a higher than 2-fold induction in 50% of the β-catenin target genes in the Mgp-/- aortae with 28 genes showing a statistically significant change (Table 3; p<0.05) and 16 additional genes showing a trend towards induction (p>0.05). Importantly, many of the β-catenin target genes significantly upregulated in the Mgp-/- arterial tissue are related to osteochondrogenic transformation or VSMC de-differentiation (Table 3). These results indicate β-catenin signaling is activated in the absence of MGP and that its activation may contribute to chondrogenic transformation in VSM.

**Table 3:** Changes in expression of Wnt/β-catenin target genes in *Mgp-/-* compared to wild-type aortic tissue.

**Gene**	**Protein**	**Fold change**	**p-value**	**Function**
Abcb1a	Transmembrane glycoprotein	6.41**	0.005	ATP-binding cassette
Abr	Aryl-hydrocarbon receptor	3.04*	0.043	Transcription factor
Birc5	Survivin	3.67***	0.001	Inhibitor of apoptosis
Cacna2d3		3.02*	0.035	Calcium channel
Cd44		2.93**	0.008	Receptor for ECM
Cdkn2a	Cyclin-dependent kinase inhibitor	5.32*	0.016	Cell cycle
Cdon	Cell adhesion molecule	2.47**	0.007	Shh receptor
Cubn	Cobalamin and vitamin B12 receptor	2.58*	0.013	
Enpp2	Autotaxin	5.32***	0.001	Lysophospholipase
Foxn1	Forkhead box N1	6.30*	0.026	Cell growth and proliferation
Mmp9	Matrix metalloproteinase 9	4.16*	0.036	VSMC migration/proliferation [63]
Nanog		13.94*	0.041	“stem-like” phenotype
Tcf4		3.89***	0.001	Transcription factor
***Chondro/Osteogenic differentiation***				
Dkk1	Dickkopf 1	9.85**	0.007	Wnt inhibitor, negative regulator of osteogenesis [64]
Fgf9	Fibroblast growth factor 9	4.14*	0.036	Chondrocyte hypertrophy [65,66]
Gdf5	Growth differentiation factor 5	5.02*	0.046	Osteogenic differentiation [67,68]
Met	Receptor for hepatocyte growth factor	2.00*	0.028	Osteogenic transformation in VSMC, bone/cartilage formation [69,70]
Mmp2	Matrix metalloproteinase 2	3.48**	0.010	Required for mineralization in VSMC [71]
Plaur	Plasminogen activator, urokinase (uPAR) receptor	3.84*	0.015	VSMC de-differentiation in [72]
T	Brachyury	3.38*	0.020	Chondrogenic differentiation [73]
Wisp1	WNT1-inducible signaling protein 1	4.30**	0.008	Osteoblast differentiation, represses chondrogenesis [74]
***Endothelial genes***				
Cdh1	E-cadherin	6.19***	0.001	
Dlk1	Delta-like 1 homolog	5.84*	0.038	Non-canonical Notch ligand, inhibits angiogenesis [75]
Nrcam	Neuron-glia-CAM-related cell adhesion molecule	16.14***	0.001	Cell communication, angiogenesis [76]
***Down-regulated genes***				
Ccnd2	Cyclin D2	-1.39*	0.040	Proliferation
Fzd7	Frizzled 7	-4.05*	0.043	Non-canonical β-catenin signaling [77]
Gja1	Connexin 43	-2.99*	0.013	Supports bone formation [78–80]
Igf2	Insulin-like growth factor 2	-3.49*	0.047	Enhances osteogenic capacity of hMSCs [81]

Aortic tissue from 4.5 week old animals was analyzed using a qRT-PCR-based microarray. N=6. * p<0.05; ** p<0.01; *** p<0.001.

### Chondrogenic transformation of VSMCs in vitro: activation of β-catenin signaling

**Figure 2:**
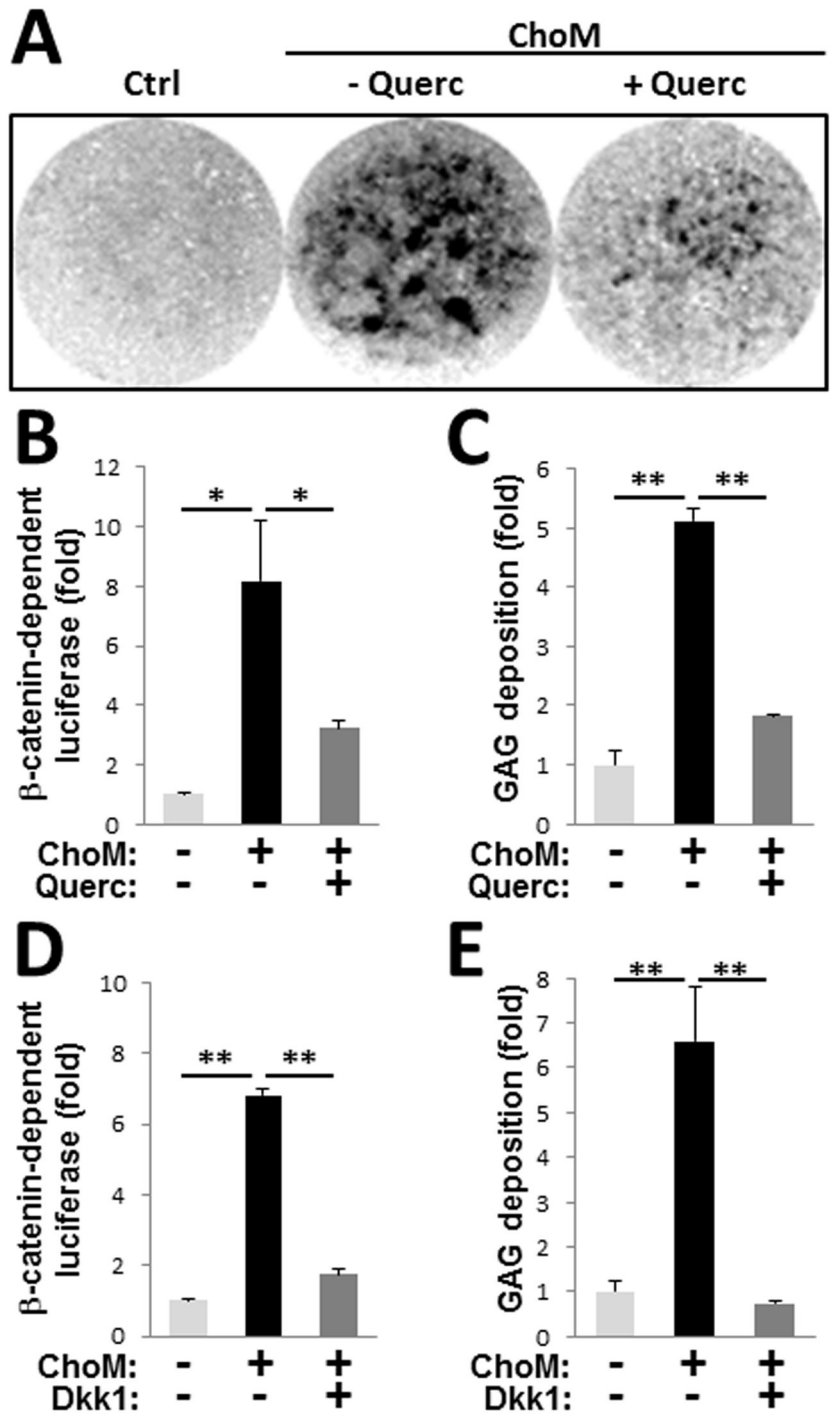
Inhibition of β-catenin signaling attenuates chondrogenic transformation of cultured VSMCs. Rat A10 VSMCs stably-expressing a β-catenin-responsive luciferase transgene were induced to undergo chondrogenesis in high-density micromass culture in chondrogenic medium (ChoM) containing TGF-β3. **A**, VSMCs form cartilaginous GAG-positive nodules as detected by Alcian blue stain, in ChoM (- Querc). In the presence of 50 µmol/L Quercetin (ChoM + Querc) both the number and size of the nodules is reduced. **B**-**E**, Expression of β-catenin-dependent luciferase reporter (**B**,**D**) and deposition of GAG-rich matrix, quantified by extraction of Alcian blue stain normalized to cell number (**C**,**E**), in VSMCs cultured in ChoM in the presence or absence of quercetin (**B**,**C**) or the β-catenin signaling pathway inhibitor Dkk1 (**D**,**E**). N=4.

To determine whether activation of β-catenin signaling directly associates with chondrogenic transformation of VSM, in contrast to being potentially induced by systemic factors in the *Mgp-/-* mice, we employed an in vitro system in which the A10 rat aortic VSMC line or primary mouse VSMCs (P2-3) were induced to undergo chondrogenic transformation in high-density micromass cultures stimulated by dexamethasone and TGF-β3 [43]. Within two weeks in culture, both rat and mouse VSMCs underwent chondrogenic transformation, evidenced by the formation of characteristic glycosaminoglycan (GAG)-rich cartilaginous nodules (Figure 2A). This was accompanied by activation of β-catenin signaling, detected by increased expression of a β-catenin-dependent luciferase transgene in A10 cells (Figure 2B, 8.16 ±2.02-fold, p<0.05).

### Chondrogenic transformation of VSMCs in vitro: attenuation by quercetin

Quercetin has been characterized as a potent inhibitor of β-catenin signaling, and in VSMCs quercetin stabilizes the contractile phenotype [25]. Here we show that quercetin significantly attenuated the activation of β-catenin signaling in chondrogenic micromass cultures of A10 cells (Figure 2B) and also significantly reduced chondrogenic differentiation in these cells as evidenced by a reduction in GAG-positive matrix detected with Alcian Blue stain (Figure 2C). To determine whether the observed inhibition of β-catenin signaling caused the reduction in GAG synthesis, we treated chondrogenic micromass cultures with recombinant Dkk1, a known inhibitor of the canonical β-catenin pathway [44]. Dkk1 prevented both increased expression of the β-catenin-dependent luciferase reporter (Figure 2D) and deposition of cartilaginous GAG matrix by A10 VSMCs (Figure 2E), demonstrating a critical role for activation of β-catenin signaling in the chondrogenic transformation of VSMCs in vitro.

**Figure 3:**
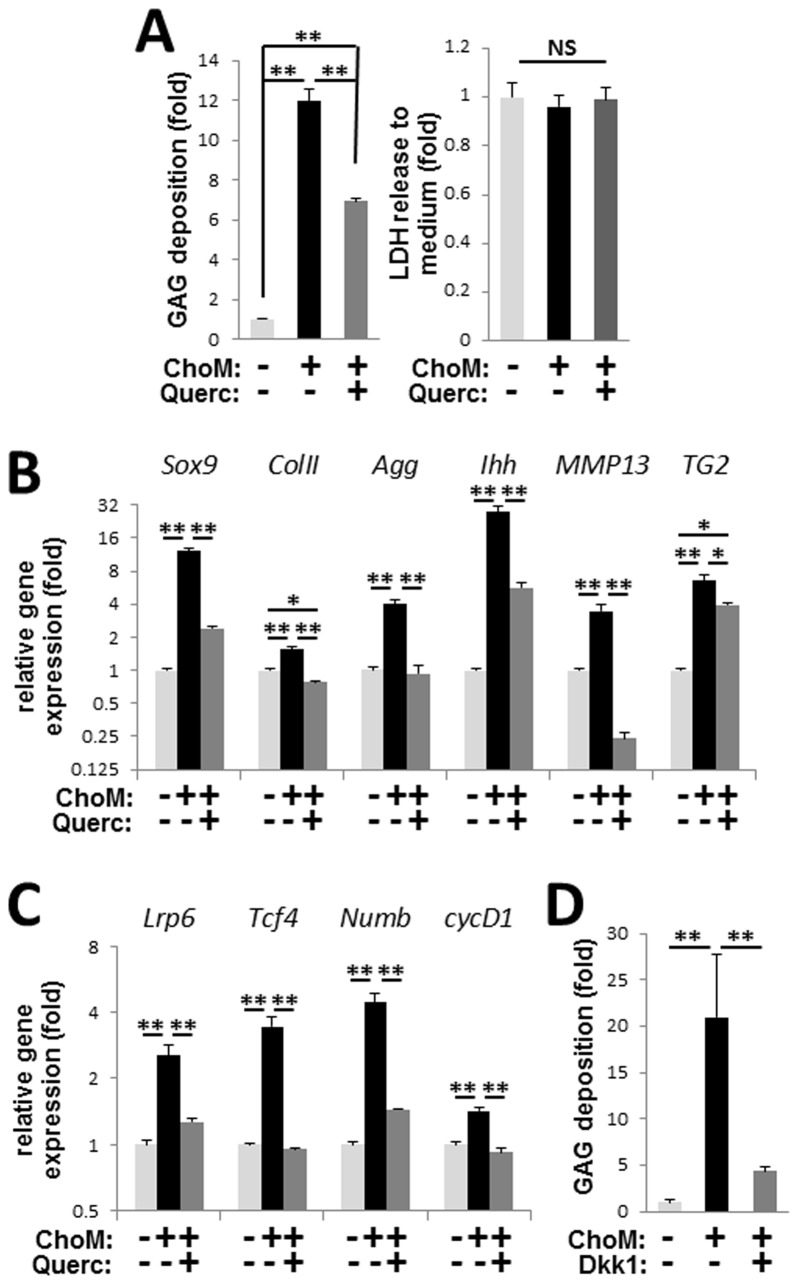
Quercetin attenuates chondrogenic transformation and β-catenin activation in primary VSMCs. **A**, Quercetin reduces deposition of GAG-positive matrix in VSMCs induced to undergo chondrogensis in high-density micromass culture in chondrogenic medium (ChoM). Quantitation of GAG deposition (*left graph*) and cell death, detected by LDH release into the culture medium (*right graph*), in the presence or absence of 50 µmol/L quercetin. N=4. **B**-**C**, Real-time PCR analysis of markers of chondrogenesis (**B**) and β-catenin target genes (**C**) in VSMC micromass cultures treated with ChoM and quercetin as indicated. N=4. **D**, Quantitation of GAG deposition by VSMC micromass cultures treated with ChoM in the presence or absence of 0.5 µg/mL Dkk1. N=4.

Similar to its effects on the A10 VSMC line, quercetin significantly reduced GAG deposition by primary mouse aortic VSMCs treated with chondrogenic medium in micromass culture without affecting cell survival as evidenced by unchanged levels of LDH activity in the culture medium (Figure 3A). Analysis of stage-specific markers of chondrogenesis using qRT-PCR showed that quercetin prevented induction of both early (Sox9, collagen type II, and aggrecan) and late (Ihh, MMP13, and TG2) markers (Figure 3B), identifying this flavonoid as a potent inhibitor of chondrogenic transformation in VSMCs and suggesting the potential therapeutic value of quercetin in preventing chondrogenic transformation of VSM in vivo. In addition, qRT-PCR analysis revealed that the expression of four endogenous β-catenin target genes induced during chondrogenic transformation of VSMCs was prevented by quercetin indicating inhibition of β-catenin signaling (Figure 3C). Further, treatment of the primary VSMC micromasses with the β-catenin pathway inhibitor Dkk1 blocked GAG-rich matrix deposition (Figure 3D), mimicking the effect of quercetin and implicating the β-catenin signaling pathway as a major target of quercetin-mediated inhibition of chondrogenic transformation in cultured VSMCs.

### Quercetin reduces calcium accrual in Mgp-/- aortae

**Figure 4:**
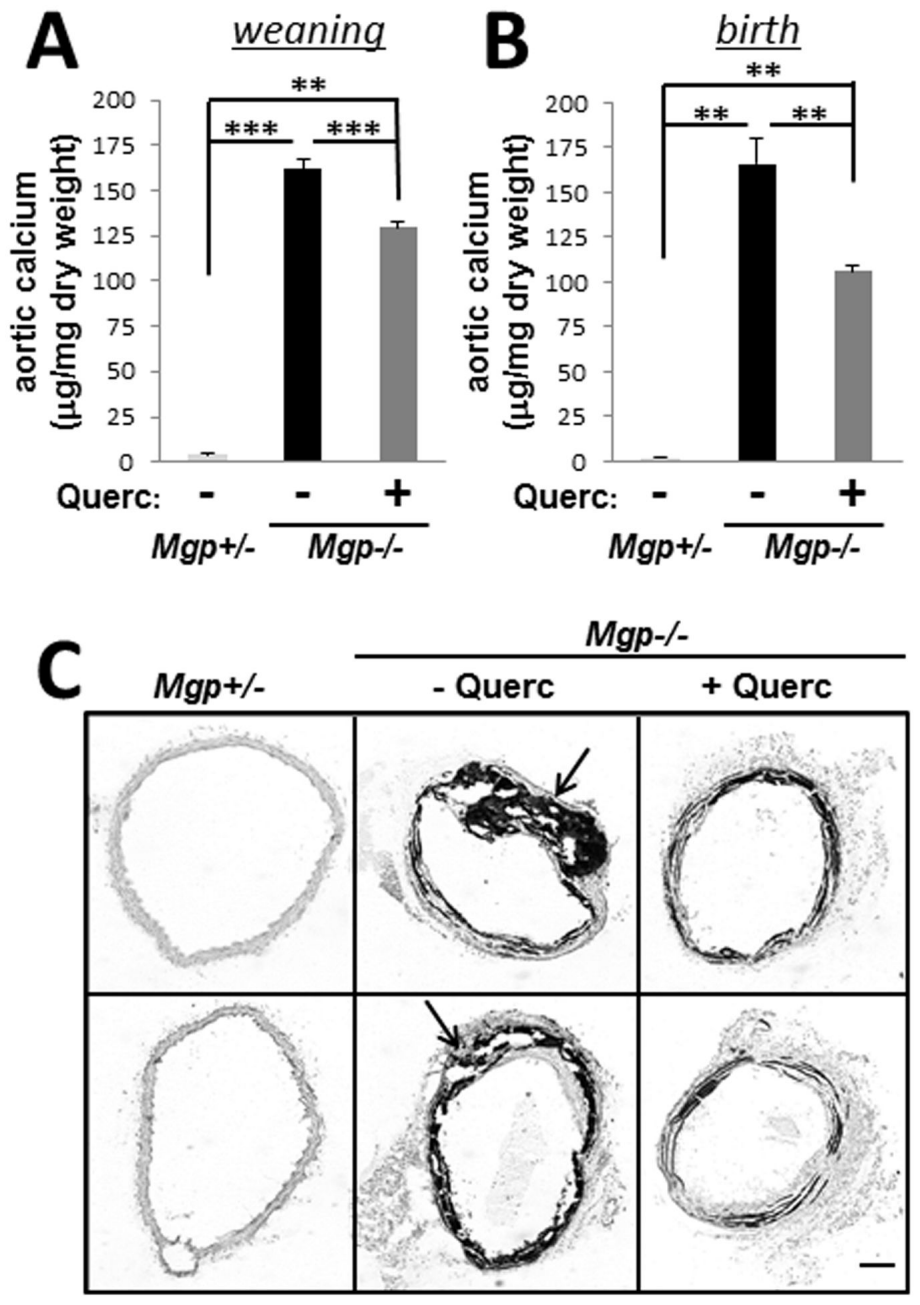
Quercetin reduces calcium accrual in *Mgp-/-* mice. **A**-**B**, Total calcium in aortic tissue from 5 week old untreated *Mgp-/*- mice (N=8) or *Mgp-/*- mice treated with 0.02% w/w dietary quercetin for 2 weeks after weaning (**A**; N=16) or for 5 weeks from birth through weaning (**B**; N=9). Heterzygous (*Mgp+/-*) mice served as control (N=6). **C**, Von Kossa stain for calcified matrix deposition in aortic tissue from *Mgp+/-*, untreated *Mgp-/*-, and quercetin-treated *Mgp-/*- animals. Sections from 2 representative animals are shown. Arrows denote chondrogenic metaplasia. Scale = 50 µm.

Taking into consideration that extensive calcification of chondrogenic metaplasia is characteristic for MGP-null vascular disease, we examined whether quercetin treatment alleviates calcification in MGP-deficient animals. After weaning, we treated *Mgp-/-* and *Mgp+/-* pups for 2 weeks with dietary quercetin (0.02% w/w in drinking water [30]) and analyzed total calcium content in the aortae biochemically by the o**-** *cresolphthalein* complexone method. We found that quercetin treatment associated with a significant 20.0±2.1% (p<0.001) reduction in total calcium content of Mgp-/- aortic tissue (Figure 4A), although aortic calcium levels in the quercetin-treated *Mgp-/-* animals remained significantly higher than those in heterozygous littermates. In addition, quercetin treatment associated with a significant reduction in the expression of osteogenic genes in *Mgp-/-* aortic tissue (Table 4).

**Table 4:** Changes in osteogenic gene expression in aortas from *Mgp-/-* mice treated with quercetin compared to untreated *Mgp-/-* aortic tissue.

**Gene**	**Protein name**	**Fold Down-Regulation**	**p-value**
AlpI	Alkaline phosphatase	-2.09**	0.032
Anxa5	Annexin 5	-1.90**	0.010
Bmpr1b	BMP receptor, type 1B	-2.94**	0.006
Col14a1	Collagen type XIV, alpha 1	-4.28**	0.008
Col1a2	Collagen type I, alpha 2	-3.18**	0.043
Col4a2	Collagen type IV, alpha 2	-2.63**	0.035
Col6a1	Collagen type VI, alpha 1	-2.53**	0.048
Col6a2	Collagen type VI, alpha 2	-3.76**	0.015
Fgf3	Fibroblast growth factor 3	-6.78**	0.002
Fgfr1	Fibroblast growth factor receptor 1	-2.19**	0.014
Itga3	Integrin alpha 3	-2.03**	0.027
Itgav	Integrin alpha V	-3.17**	0.002
Mmp8	Matrix metalloproteinase 8	-4.20**	0.051
Smad1	MAD homolog 1	-1.48**	0.017
Tgfb3	Transforming growth factor, beta 3	-2.77**	0.001
Tgfbr1	Transforming growth factor receptor, beta 1	-3.19**	0.043
Col10a1	Collagen type X, alpha 1	-8.07**	0.105
Mmp10	Matrix metallopeptidase 10	-7.06**	0.145

Tissue from 4.5 week old animals was analyzed using a qRT-PCR-based microarray N=5. *, p<0.05; ** p<0.01.

We hypothesized that quercetin may prevent the *de novo* formation of calcifying cartilaginous metaplasia but not reverse calcification already present in 3 week old animals at weaning. Therefore, to affect younger animals we treated newborn *Mgp-/-* mice with quercetin for 5 weeks (via the lactating moms until weaning and then in the drinking water as described above) and analyzed calcium accrual in these mice compared to *Mgp+/-* littermates. Quercetin treatment from birth produced a 36.3±1.7% (p<0.01) reduction in total calcium content of Mgp-/- aortic tissue (Figure 4B), almost twice what was observed in animals treated with quercetin for only 2 weeks. However, calcium levels in the quercetin-treated *Mgp-/-* aortae still remained significantly elevated. To examine this phenomenon further, localization of calcium phosphate deposits in the arterial walls was detected with von Kossa staining (Figure 4C). Excessive calcium phosphate deposits aligning with elastic lamellae were present in both untreated and quercetin-treated *Mgp-/-* mice, suggesting that elastocalcinosis accounts for the remaining 64% of calcium deposits in quercetin-treated mice thus identifying elastocalcinosis as a major contributor to MGP null vascular disease. In contrast, the massive calcified zones corresponding to cartilaginous metaplasia were absent in quercetin-treated Mgp-/- mice, indicating that in vivo chondrogenic transformation of VSM is sensitive to quercetin treatment.

### Dietary quercetin alleviates cartilaginous metaplasia

**Figure 5:**
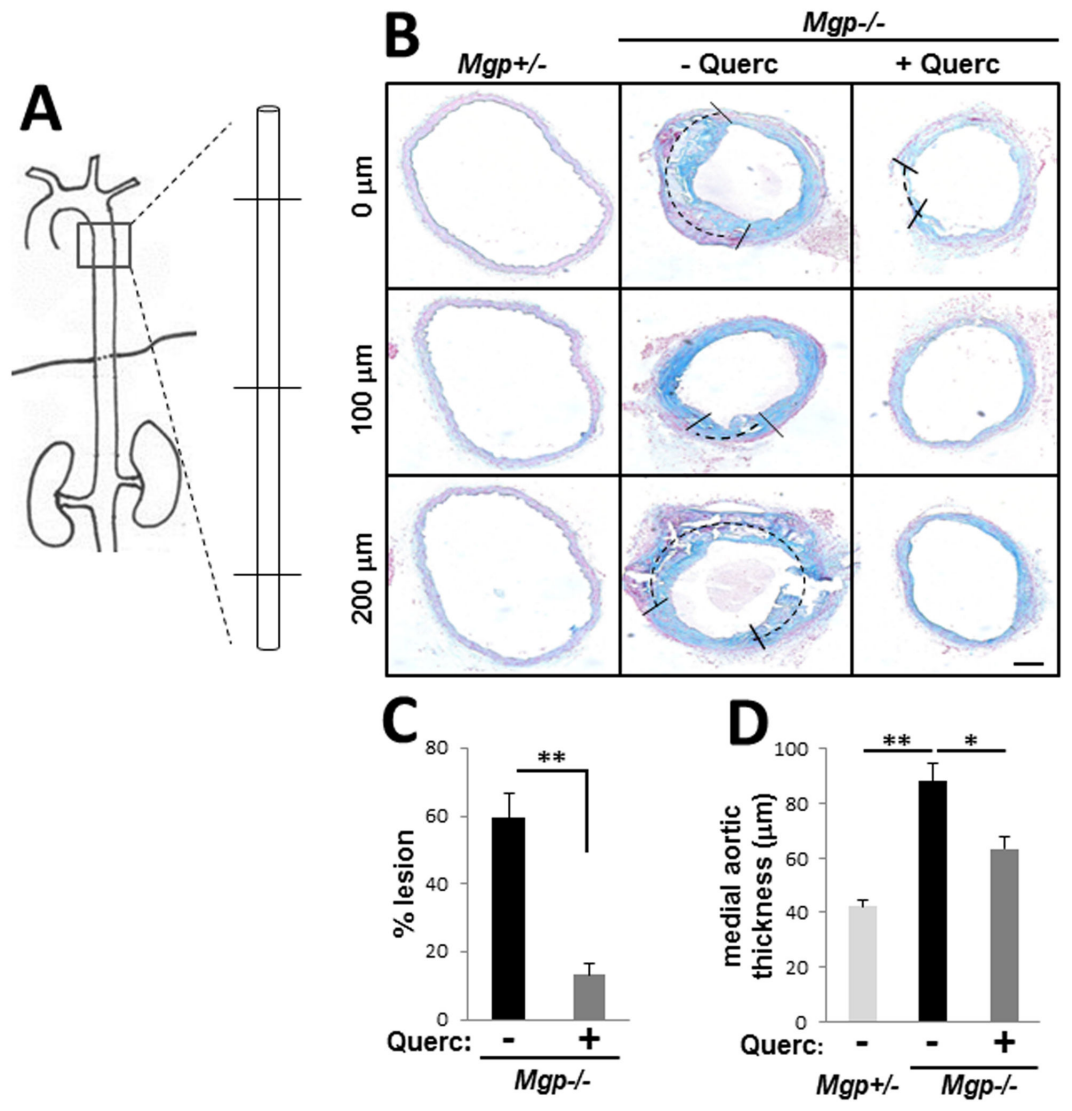
Quercetin prevents chondroplasia and improves vessel morphology in *Mgp-/-* mice. **A**, Serial sections spaced 100 µm apart through a 1 mm segment of descending aorta from each animal were analyzed. **B**, Representative images of serial sections from *Mgp+/-* (N=3), untreated *Mgp-/*- (N=5), and quercetin-treated *Mgp-/*- (N=6) animals stained with Alcian blue. Dashed lines denote the proportional presence of chondroplastic lesions in the vessel wall. Scale = 50 µm. **C**-**D**, Quantitative analysis of the serial sections of aortae show that quercetin diet reduces the percentage of vessel circumference occupied by chondrogenic lesions (**C**) and the cross-sectional thickness of the medial aorta (**D**).

We next analyzed the reduction of cartilaginous metaplasia by quercetin in more detail. Aortae were collected from *Mgp-/-* mice treated with quercetin for 4.5 weeks and vessel wall morphology was analyzed along a 1mm long aortic segment using serial tissue sections collected 100 µm apart (Figure 5A). Quercetin diet prevented cartilaginous metaplasia (indicated by dashed lines) and improved gross morphology of the *Mgp-/-* aortic wall (Figure 5B). Quantitatively, quercetin treatment significantly reduced the proportion of cartilaginous lesions in the circumference of vessel wall from 59.6±7.3% in the untreated Mgp-/- mice (N=5) to 13.3±3.1% in the quercetin treated animals (N=6, p<0.01) (Figure 5C). Accordingly, we detected an approximately 30% reduction in arterial wall thickness (64.2±5.0 µm in quercetin-treated Mgp-/- mice compared to 90.1±9.9 µm in untreated *Mgp-/-* animals), rendering this parameter similar to that of the control aortae (*Mgp+/-*, N=3) (Figure 5D).

Further, qRT-PCR analysis revealed that expression of cartilaginous extracellular matrix proteins and transcription factors associated with chondrogenesis [45,46] were increased in *Mgp-/-* animals, and this increase was significantly attenuated in quercetin-treated *Mgp-/-* animals (Table 5). Of note, both early and late markers of chondrogenesis were attenuated by quercetin, implying that the prevention of chondrogenic transformation of VSM by this flavonoid in vivo is similar to its action in vitro.

**Table 5:** Changes in expression of genes related to chondrogenic differentiation in *Mgp-/-* versus wild-type and in quercetin-treated *Mgp-/-* (Mgp-/- +Q) versus untreated *Mgp-/-* aortic tissue from 4.5 week old animals analyzed by qRT-PCR-based microarray.

**Gene**			**Protein**	**Fold change in expression**					
				*Mgp-/-* to wild-type	p-value		Mgp-/- +Q to *Mgp-/-*	p-value	
***Extracellular matrix proteins***									
Col11a1			Collagen, type XI	4.34***		0.005	-3.79***		0.006
Col12a1			Collagen, type XII	9.86***		0.029	-3.94***		0.031
Col1a1			Collagen, type I	7.28***		0.034	-2.58***		0.143
Col2a1			Collagen, type II	1152.89***		0.015	-22.74***		0.020
Comp			Cartilage oligomeric matrix protein	75.92***		0.031	-3.26***		0.058
Dmp1			Dentin matrix protein 1	12.19***		0.048	-4.52***		0.070
Fn1			Fibronectin 1	4.31***		0.031	-1.94***		0.138
Vcam1			Vascular cell adhesion molecule 1	11.62***		0.015	-2.17***		0.073
Mmp2			Matrix metalloproteinase 2	3.48***		0.010	-2.01***		0.028
***Transcription factors***									
Msx1		Homeobox, msh-like 1		2.85***		0.003	-3.66***		<0.001
Runx2		Runt related transcription factor 2		8.81***		<0.001	-2.40***		0.002
Sox9		SRY-box containing gene 9		7.95***		0.002	-2.24***		0.028
Twist1		Twist homolog 1 (Drosophila)		1.69***		0.040	-2.19***		0.027
Vdr		Vitamin D receptor		5.38***		0.008	-1.03***		0.740

N=5. *, p<0.05; ** p<0.01; *** p<0.001.

### Dietary quercetin blocks nuclear accumulation of β-catenin protein in Mgp-/- aortae

**Figure 6:**
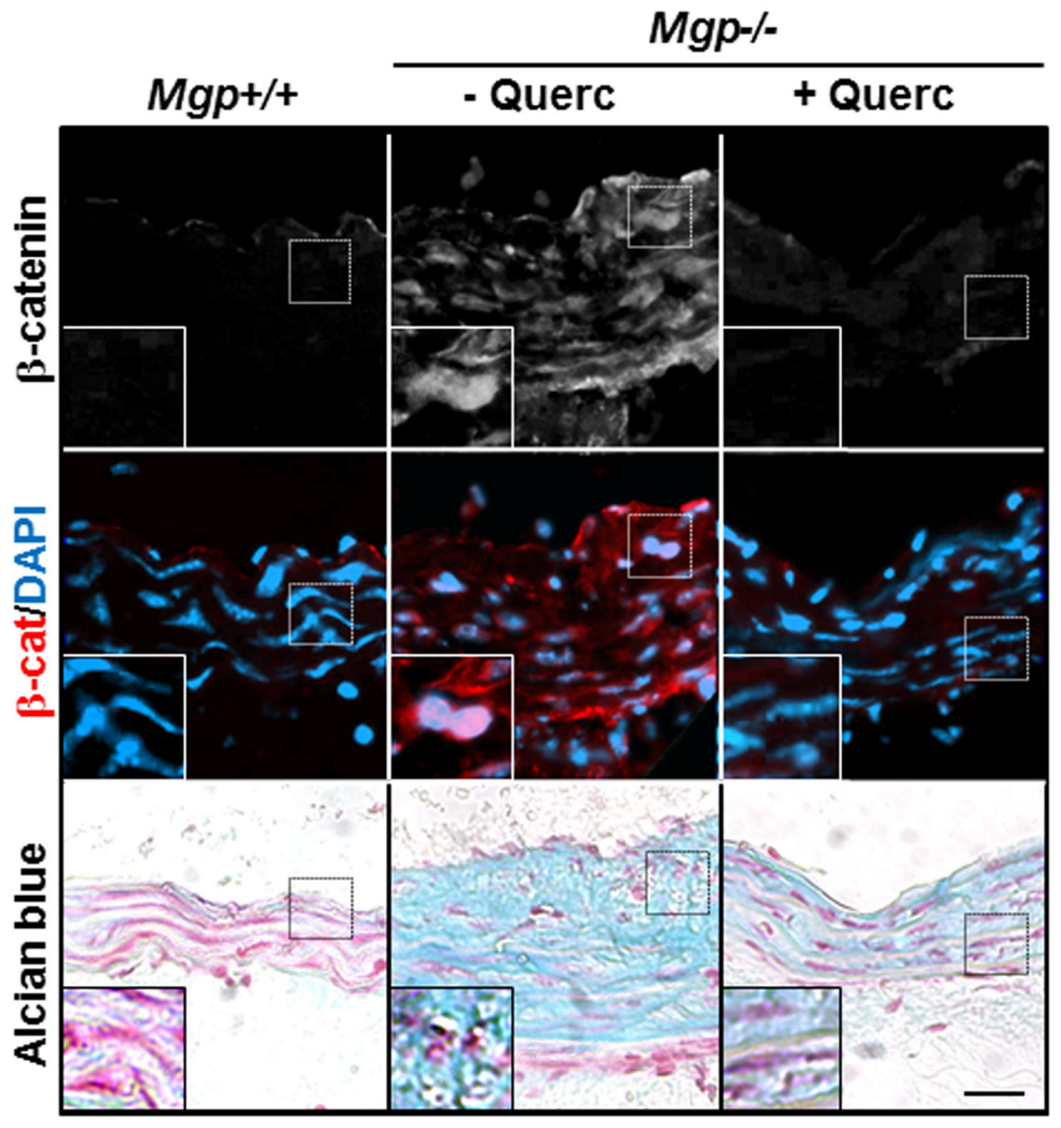
Quercetin blocks accumulation and nuclear localization of β-catenin protein in *Mgp-/-* aortae. Immunostaining for β-catenin (white, red) [nuclei counterstained with DAPI (blue)] and Alcian blue staining for GAG deposition on adjacent sections of aortae from *Mgp+/+*, untreated *Mgp-/*-, and quercetin-treated *Mgp-/*- animals shows that β-catenin protein is detected only in untreated *Mgp-/*- arterial tissue. Scale = 30 µm. Inset panels show higher magnification of representative nuclei, demonstrating co-localization of β-catenin with DAPI in the *Mgp-/*- aorta in rounded chondrocyte-like cells surrounded by cartilaginous GAG-rich matrix.

Elaborating on our findings that the inhibition of chondrogenesis in cultured VSMCs associates with inhibition of β-catenin signaling, we tested whether quercetin similarly affects this signaling conduit in MGP null arterial tissue. Accumulation and nuclear localization of β-catenin protein, indicative of activation of this pathway [47], were examined in wild-type and *Mgp-/-* aortic tissue. In wild-type aortic tissue with normally inactive β-catenin signaling [48], β-catenin protein was not observed (Figure 6, *Mgp+/+*). In contrast, β-catenin protein was readily detected by immunofluorescence in the aortae of 5 week old *Mgp-/-* mice in which foci of alcian blue-positive cartilaginous metaplasia develop in the tunica media (Figure 6, *Mgp-/-*, - Querc). Further, in the quercetin-treated Mgp-/- mice we did not detect any accumulation of β-catenin protein in the vessel media (Figure 6, *Mgp-/-* + Querc), suggesting that the in vivo morphological effects of quercetin correlated with attenuation of β-catenin signaling. To further clarify the relationship between β-catenin activation and calcifying chondrogenic metaplasia in vivo, we determined the location of cells with active β-catenin within arterial tissue. Nuclear β-catenin protein was detected in chondrocyte-like cells, which appear as rounded cells surrounded by alcian blue positive GAG-rich matrix, in the *Mgp-/-* aortae (Figure 6, insets), indicating that activation of the β-catenin signaling associates with chondrogenic transformation in VSM in vivo.

## Discussion

Data from this study demonstrate that MGP null vascular disease associates primarily with chondrogenic but not osteogenic transformation of VSM as we did not detect expression of the key regulator of osteogenesis osterix or induction of Msx2 in calcified *Mgp-/-* aortae. Similarly, a previous study suggested chondrogenic transformation as a major cell fate of the *Mgp-/-* VSM [13]. Of note, stimulation of the β-catenin signaling pathway can promote chondrogenic differentiation of mesenchymal cells in a Sox-9-dependent manner [49] and in our system we also detected an increase in both β-catenin activity and Sox-9 expression. Although activation of canonical β-catenin signaling is broadly linked to cardiovascular disorders [35,48,50], our study is the first to our knowledge to demonstrate activation of this signaling pathway in VSM undergoing chondrogenic transformation in vitro and in vivo.

The origin of increased β-catenin activity in the *Mgp-/-* aortae deserves further inquiry. The canonical Wnt ligands expressed in the *Mgp-/-* aortae are present at very low levels, in contrast to the robust induction of Wnt3a and Wnt7a reported in the diabetic model. The observed dramatic reduction in expression of the β-catenin pathway antagonist sclerostin may contribute to activation of the pathway in the *Mgp-/-* model, and non-canonical agonists of this signaling may also play a role. For example, enzyme transglutaminase 2 (TG2), an important regulator of β-catenin in calcifying VSM [24,51], accumulates in *Mgp-/-* vessels in vivo [42] and is induced in VSMCs undergoing chondrogenic differentiation in vitro (Figure 3B). Taking into account that quercetin inhibits TG2 via direct binding [12] it is possible that modulation of TG2 enzymatic activity is central in chondrogenic metaplasia of VSM and its prevention. Current studies are underway to address this possibility.

Although quercetin drastically attenuated chondrogenic metaplasia and its calcification, and intercepted chondrogenic transformation of VSM as evidenced by attenuated expression of pro-chondrogenic master regulators Sox9, Twist1, and Runx2, the overall calcium accrual in *Mgp-/-* blood vessels was only partially alleviated by this flavonoid. About one-half of total mineral remained as deposits along the elastic lamellae, supporting the model that elastocalcinosis may precede chondrocyte-like transformation in VSM [52] and consistent with the model in which MGP acts as a direct inhibitor of tissue calcification by binding to small calcium phosphate precipitates and/or the elastic lamellae, thereby preventing nucleation and growth of mineral crystals (reviewed in [53]) This finding challenges the perception of MGP-null arterial disease as primarily a product of ectopic chondrogenesis resulting in calcification [13], and emphasizes the importance of elastocalcinosis in this pathology.

Attenuation of chondrogenic transformation in cultured VSMCs by quercetin associated with inhibition of β-catenin signaling, and in the *Mgp-/-* aortae quercetin treatment blocked accumulation and nuclear localization of β-catenin protein, supporting a role for this pathway in the phenotypic instability of VSM. While inhibition of β-catenin signaling may be one of the major mechanisms of quercetin action in this system, the possible contribution of other known quercetin activities including anti-inflammatory and anti-oxidative effects cannot be excluded. However, the equally low cell proliferation in *Mgp-/-* and wild type vessels demonstrated in this study and earlier [13] indicate that cartilaginous metaplasia is not linked to hyperproliferation and suggest a limited role for the anti-proliferative activity of quercetin. While the exact mechanisms whereby quercetin achieves a reduction in cartilaginous metaplasia are not definitively proven, the data support a role for inhibition of β-catenin signaling as a significant part of the action.

In conclusion, this study adds to the growing understanding of the complexity of MGP-mediated effects on vascular tissue by adding the β-catenin signaling pathway to the MGP-modulated cellular network that already includes BMP [54] and Notch [55] conduits, and by showing that MGP may regulate elastocalcinosis even when chondrogenic transformation is suppressed. In addition, the presented studies contribute to the mechanistic understanding of the beneficial effects of quercetin in cardiovascular disease and identify a potential therapeutic approach to the calcifying cartilaginous metaplasia of VSM with quercetin that stands out as a pharmacological tool with remarkably wide safety margin. Of note, the effects of quercetin delivered in drinking water or via breast milk are similar in *Mgp-/-* mice indicating that dietary supplement of quercetin in breastfeeding females should be considered carefully for possible effects on infants.
